# Chenodeoxycholic Acid Modulates Bile Acid Synthesis Independent of Fibroblast Growth Factor 19 in Primary Human Hepatocytes

**DOI:** 10.3389/fendo.2020.554922

**Published:** 2021-02-22

**Authors:** Helene Johansson, Jonas Nørskov Søndergaard, Carl Jorns, Claudia Kutter, Ewa C. S. Ellis

**Affiliations:** ^1^ Division of Transplantation Surgery, Department of Clinical Science, Intervention and Technology (CLINTEC), Karolinska Institutet, Stockholm, Sweden; ^2^ Department of Transplantation, Karolinska University Hospital, Stockholm, Sweden; ^3^ Science for Life Laboratory, Department of Microbiology, Tumor and Cell Biology, Karolinska Institutet, Stockholm, Sweden

**Keywords:** bile acid metabolism, cholesterol 7-alpha hydroxylase, liver, transcription, nuclear receptors, RNA sequencing

## Abstract

Bile acids (BAs) are detergents essential for intestinal absorption of lipids. Disruption of BA homeostasis can lead to severe liver damage. BA metabolism is therefore under strict regulation by sophisticated feedback mechanisms. The hormone-like protein Fibroblast growth factor 19 (FGF19) is essential for maintaining BA homeostasis by down regulating BA synthesis. Here, the impact of both FGF19 and chenodeoxycholic acid (CDCA) on primary human hepatocytes was investigated and a possible autocrine/paracrine function of FGF19 in regulation of BA synthesis evaluated. Primary human hepatocytes were treated with CDCA, recombinant FGF19 or conditioned medium containing endogenously produced FGF19. RNA sequencing revealed that treatment with CDCA causes deregulation of transcripts involved in BA metabolism, whereas treatment with FGF19 had minor effects. CDCA increased FGF19 mRNA expression within 1 h. We detected secretion of the resulting FGF19 protein into medium, mimicking *in vivo* observations. Furthermore, medium enriched with endogenously produced FGF19 reduced BA synthesis by down regulating CYP7A1 gene expression. However, following knockdown of FGF19, CDCA still independently decreased BA synthesis, presumably through the regulatory protein small heterodimer partner (SHP). In summary, we show that in primary human hepatocytes CDCA regulates BA synthesis in an FGF19-independent manner.

## Introduction

Bile acids (BAs), synthesized from cholesterol by the liver, enter the enterohepatic circulation to function as detergents in the intestine for absorption of dietary lipids. This efficient system ensures that the majority of BA returns from the intestine to the liver and only 1–5% of the total pool need to be replaced by newly synthesized BAs on a daily basis (1–3). BA homeostasis is essential, and disturbance can cause severe complications such as malabsorption of nutrients and excess BAs may cause cell injury that in turn leads to liver fibrosis and cirrhosis. Synthesis, transport and circulation of BAs are therefore under rigorous control (1–5). BAs act as signaling molecules to the nuclear receptor Farnesoid X receptor (FXR) in both intestine and liver and FXR in turn induces regulatory pathways (5–7). In recent years, the FXR induced hormone-like protein Fibroblast growth factor 19 (FGF19) has been of particular interest in respect to BA regulation. Upon activation by re-absorbed BAs, intestinal FXR induces FGF19 expression. FGF19 is released to the portal blood stream and when reaching the liver it signals to suppress the rate-limiting enzyme in BA synthesis, cholesterol 7-alpha hydroxylase (CYP7A1) (8–10). FGF19 expression is low or absent in healthy liver and this is mirrored in cultures of primary human hepatocytes. The liver starts expressing FGF19 and circulating levels increase under pathophysiological conditions for example when bile flow from the liver is restricted. Furthermore, primary human hepatocytes express FGF19 when treated with chenodeoxycholic acid (CDCA), the primary BA with highest affinity for FXR in humans (3, 8, 9, 11, 12). We have previously established circulating levels of FGF19 under physiological conditions and demonstrated that FGF19, unlike BAs, do not display a gradient over the liver (13). With this study we aimed to gain a better understanding of how FGF19 affects primary human hepatocytes and in particular how it affects BA synthesis. We evaluated the impact of CDCA and recombinant FGF19, within the physiological range of concentrations, on primary human hepatocytes in respect to BA synthesis. The concentrations used were kept around the established postprandial levels in the portal circulation of FGF19 (approximately 400 pg/ml) and CDCA (approximately 10 µM) (10, 13, 14). We further investigated the effect of conditioned medium with endogenously produced FGF19 on primary human hepatocytes. A possible autocrine/paracrine function of FGF19 was evaluated by knockdown of FGF19 and inhibition of bile acid synthesis by CDCA was assessed. Differential expression in primary human hepatocytes by RNA sequencing following treatment with CDCA, recombinant FGF19 or endogenously produced FGF19 was investigated. In short, we demonstrated that although CDCA rapidly induced FGF19 in primary human hepatocytes, and conditioned medium suppressed CYP7A1, CDCA still efficiently downregulated CYP7A1 following FGF19 knockdown. Thus, CDCA regulate BA synthesis independently of FGF19.

## Materials and Methods

### Isolation of Primary Human Hepatocytes

Primary human hepatocytes were isolated from patients undergoing liver resection, from extirpated livers or from donor livers rejected for transplantation. Information about the livers in each experiment is summarized in **Table 1**. The isolation procedure followed a three-step perfusion technique developed by Berry and Friend (15) and optimized for primary human hepatocytes by Strom et al. (16). Cells were cultured on matrigel derived from Engelbreth-Holm-Swarm sarcoma (Sigma-Aldrich, St. Louis, MO) and in William’s E medium (Invitrogen, Waltham, MA) supplemented with 20 mM HEPES (Lonza, Basel, Switzerland), 2 mM glutamine (Sigma-Aldrich, St. Louis, MO), 10 nM insulin, 100 nM dexamethasone, 0.01 M gentamicin (Lonza, Basel, Switzerland) and 55 nM amphotericin B, for five days at 37°C in 5% CO2.

**Table 1:** Demographics.

Experiment	N	Analysis	Donor ID	Gender (F/M)	Age (year)Median (min-max), Mean (±SEM)	Cell viability % Median (min-max), Mean (±SEM)	Diagnosis
**FGF19/CDCA 24 h**	13	qPCR (n = 13) ELISA (n = 13) BA analysis (n = 5)^#^	13,38^#^,183^#^, 188,189,192, 194,195,198, 359,414^#^,425^#^, 432^#^	8/5	47.0 (0–77) 49.1 (±7.4)	77.0 (62–94) 76.6 (±2.4)	Donor (n = 5), CRC (n = 3), CCC (n = 2), Cholangitis (n = 1), Hyperoxaluria (n = 1), Neuroendocrine tumor (n = 1)
**FGF19/CDCA 6 h**	10	qPCR (n = 10) ELISA (n = 10) BA analysis (n = 5)^#^ RNA sequencing (n = 3)^†^	16^†^,38^#^,207, 210^#^,224^†^,226^†^, 359,414^#^,425^#^, 432^#^	3/7	46.0 (0–76) 39.4 (±10.6)	80.0 (71–94) 81.9 (±2.5)	Donor (n = 3), CRC (n = 1), CCC (n = 1), HCC (n = 2) Hyperoxaluria (n = 1), Alagille syndrome (n = 1), MSUD (n = 1)
**Endogenous FGF19**	10	qPCR (n = 10) ELISA (n = 10) BA analysis (n = 5)^#^ RNA sequencing (n = 3)^†^	16^†^,38^#^,39^†^, 219,375^#†^,414^#^, 425^#^,432^#^,444, 445	3/7	32.0 (0–76) 38.3 (±9.4)	80.0 (66–86) 76.9 (±2.0)	Donor (n = 5), CRC (n = 2), CCC (n = 1), MSUD (n = 1), Unknown (n = 1)
**Time course**	3	qPCR (n = 3) ELISA (N = 3)	414,425,432	1/2	73.0 (27–76) 58.7 (±15.9)	80.0 (71–86) 79.0 (±4.4)	Donor (n = 2), CCC (n = 1)
**siRNA**	3	qPCR (n = 3) ELISA (n = 3)	456,458,461	2/1	57 (12–76) 48.8 (±19.9)	77.0 (72–87) 78.7 (±4.4)	Donor (n = 1), CRC (n = 2)

^#^Donors used for BA analysis.

^†^Donors used for RNA sequencing analysis.

CRC, colorectal metastasis; HCC, hepatocellular carcinoma; CCC, cholangiocellular carcinoma; MSUD, maple syrup urine disease.

Characteristics and number of livers used to isolate cells for the respective experiments.

### Treatment of Primary Human Hepatocytes

Cells were kept in culture for a total of 5 days prior to harvesting and reagents were added at concentrations and time points for the different experiments as follow.

For the dose-response experiment, cells were treated with CDCA (Sigma-Aldrich, St. Louis, MO) or recombinant FGF19 (R&D systems, Minneapolis, MN) for 24 h (n = 13) or 6 h (n = 10) before harvesting, at concentrations stated.

For the time course experiment (n = 3), 10 µM CDCA was added to cultures for 10 min and up to 6 h prior to harvesting.

For the endogenous experiment (n = 10), cells were treated with 40 µM CDCA (induction medium) or regular medium without CDCA (control medium) for 6 h. After washing the cells several times with fresh medium, new medium was added and cells were kept for an additional 18 h (conditioned/control medium). The conditioned medium with all its secreted compounds was then transferred to naïve cells and treated for 24 h with 100%, 50% or 10% of conditioned or control medium (see **Figure 5A** for a layout).

A summary of livers included in the respective experiments can be found in **Table 1**.

### siRNA Gene Silencing of FGF19

Knockdown of FGF19 was performed using Silencer select siRNA and transfected by Lipofectamine RNAiMAX (ThermoFisher/Life Technologies, Carlsbad, CA). Lipofectamine/siRNA solution was prepared in Opti-MEM (Life Technologies, Carlsbad, CA) according to manufacturer’s instructions. Cells were transfected with 100 pmol siRNA (FGF19, assay ID s19355) or non-targeting negative control siRNA (cat# 4390843) for 18 h prior to co-treatment with 10 µM CDCA for 6 h (n = 3), CDCA was added directly to the existing medium.

### RNA Preparation and Quantification by qPCR

RNA was extracted using TRIzol (Invitrogen, Waltham, MA) according to manufacturer’s instructions. Quantification of mRNA was performed in triplicates with TaqMan assays on an ABI Step-One Plus instrument (Applied Biosystems, Waltham, MA). Relative mRNA expression was calculated from Ct-values against the housekeeping genes Cyclophillin A and GAPDH. TaqMan probes were purchased from Applied Biosystems (Waltham, MA): Cyclophillin A—Hs99999904_m1; GAPDH—Hs02786624_g1; FGF19—Hs00192780_m1; CYP7A1—Hs00167982.

### Quantification of FGF19 by ELISA

FGF19 concentration in cell supernatants was determined using ELISA (Human FGF19 Quantikine ELISA kit, R&D systems, Minneapolis, MN) according to the manufacturer’s instructions. Samples were analyzed in technical triplicates.

### Extraction and Quantification of Bile Acids by GC-MS

Levels of cholic acid (CA) in cell supernatants was analyzed by extraction from 1 ml supernatant as first described by Björkhem and Falk (17). In short, cell supernatant was mixed with 2,500 ng deuterium-labeled internal standard (D_2_-CDCA and D_4_-CA) and 1 M potassium hydroxide and hydrolyzed overnight at 120°C. BAs were extracted by basic ether extraction followed by acidic ether extraction, methylated with trimethylsilyl diazomethane and converted into derivates with hexamethyldisilazane–trimethylchlorosilane–pyridine. All reagents were purchased from Sigma-Aldrich (St. Louis, MO). BAs were separated and quantified against a standard curve with GC-MS (6890 Network GC system/5973 Network mass selective detector, Agilent Technologies, Santa Clara, CA), using the MassHunter Workstation software, version B.04.00/Build4.0.225.0 (Agilent Technologies, Santa Clara, CA).

### RNA Sequencing

RNA sequencing was carried out on RNA from hepatocytes treated with either 10 µM CDCA or 1000 pg/ml recombinant FGF19 for 6 h and compared to non-treated control cells (n = 3), and on hepatocytes treated with 100% conditioned or control medium (n = 3). After depleting ribosomal RNA by using Ribo-Zero Gold (Illumina, San Diego, CA), RNA sequencing libraries were prepared using the Illumina TruSeq Stranded RNA Library Prep Kit v2 (dual index) according to the manufacturer’s instruction. The quality of every cDNA library was determined on an Agilent Bioanalyzer instrument according to the manufacturer’s protocol. Library concentrations were quantified with the KAPA-SYBR FAST qPCR kit, and referenced to the provided standards (Roche, Basel, Switzerland). The sequencing run was performed with the NextSeq 500/550 High Output v2 kit (Illumina, San Diego, CA) for 150 cycles, paired end, on a NextSeq 500 instrument (Illumina, San Diego, CA). All raw data (fastq files) are deposited in ArrayExpress under accession number: E-MTAB-8627.

### Quality Control and Processing of RNA Sequencing Data

Next generation sequencing read quality was assessed with FastQC (v.0.11.5). Adaptor sequences were trimmed, and low-quality reads removed using Trimmomatic (v.0.32). Sequencing reads aligning (Bowtie2, v.2.2.9) to annotated ribosomal RNA genes were discarded. High-quality and ribosomal RNA depleted sequencing reads were aligned to the genome using TopHAT2 (v.2.0.3). Using sorted bam files (Samtools v.1.5), the number of aligned reads was counted (HTSeq-count v.0.7.2, **Table S1**). After normalization (TMM: trimmed mean of M-values, **Table S2**), a differential gene expression analysis (edgeR v. 3.3.3 in R v. 3.4.3) was performed. Significant differentially expressed genes were distinguished by a false discovery rate (FDR) under 0.05. Gene ontology analysis was performed in R (v. 3.4.3) with clusterProfiler (v. 3.6.0) and org.Hs.eg.db (v. 3.5.0). Additionally, the following dependent package versions were installed: DOSE (v. 3.4.0), AnnotationDbi (v. 1.40.0), IRanges (v. 2.12.0), S4Vectors (v. 0.16.0), BiocGenerics (v. 0.24.0), and Biobase (v. 2.38.0). Scripts for the analysis are deposited on Github (https://github.com/jonasns/FGF19).

### Statistics

Non-parametric tests were used, and graphs are presented as median with interquartile range (IQR). Wilcoxon matched-pairs signed rank test were used to assess differences between two groups and the Friedman test was used to evaluate differences between three or more groups. Dunn’s multiple comparison test was used *post-hoc* when an overall significant difference was found with the Friedman test. Differences were considered significant when p < 0.05.

## Results

### Treatment With CDCA, But Not Recombinant FGF19, Reduces BA Synthesis in Primary Human Hepatocytes

**Figure 1:**
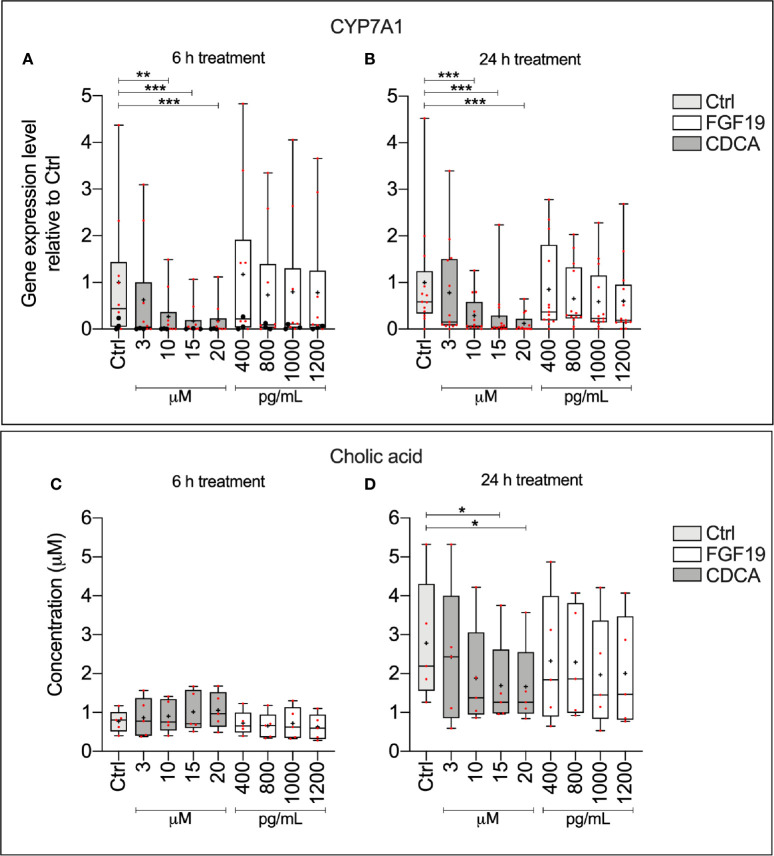
Chenodeoxycholic acid (CDCA), but not recombinant FGF19, downregulated bile acids (BA) synthesis in primary human hepatocytes. **(A, B)** CYP7A1 mRNA expression (24 h, n = 13; 6 h, n = 10) and **(C, D)** cholic acid (CA) concentration (24 h, n = 5; 6 h, n = 5) in cell medium following treatment with FGF19 or CDCA at various concentrations for 24 h or 6 h. Data is presented as box-plot showing interquartile range (IQR) (box) and min-max (bars) with median marked with a line, red dots are the individual values, black dots in **(A)** represents RNA from donors that were also used for RNA sequencing. The plus sign represents mean value. Friedman test was used to assess differences between control and treatments and Dunn’s multiple comparison test was used *post hoc*. *p < 0.05, **p < 0.01, ***p < 0.001.

To explain physiological alterations in BA synthesis, we investigated the underlying molecular responses stimulated upon FGF19 and CDCA treatment in primary human hepatocytes. We measured CYP7A1 gene expression levels and CA concentrations secreted into the cell medium from primary human hepatocytes that were treated with various concentrations of recombinant FGF19 (400–1,200 pg/ml) or CDCA (3–20 µM) for 6 h or 24 h (**Figure 1**). No significant changes in CYP7A1 gene expression levels were found upon treatment of hepatocytes with various concentrations of recombinant FGF19 at any time point when compared to untreated controls. In contrast, CYP7A1 gene expression levels were significantly lower in cultures treated with CDCA at concentrations above 3 µM for both time points compared to controls (**Figures 1A, B**). In parallel, we determined that median levels of CA in the cell supernatant was approximately two-fold lower in hepatocytes cultures after 24 h treatment with 15 µM and 20 µM of CDCA (median CA level 1.2 µM and 1.2 µM, respectively) compared to controls (median CA level 2.2 µM). No difference in CA levels in cell supernatant was observed in cultures treated with CDCA for 6 h. There was no difference in CA levels in cultures treated with recombinant FGF19 irrespective of time or concentration (**Figures 1C, D**). Our results showed that CDCA affected BA synthesis by reducing gene expression levels of the rate-limiting enzyme CYP7A1 and formation of CA in a concentration-dependent manner. No changes were observed in CYP7A1 gene expression or CA levels in cultures treated with recombinant FGF19.

### CDCA Treatment Increased FGF19 Gene Expression in Primary Human Hepatocytes and FGF19 Enriched Cell Medium Subsequently Suppressed CYP7A1 Gene Expression

**Figure 2:**
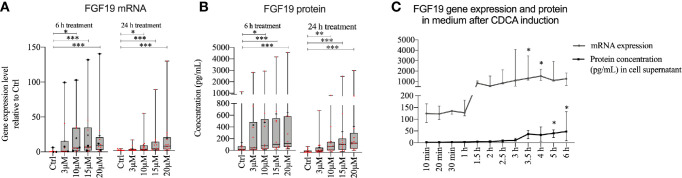
FGF19 was rapidly induced and secreted in a dose-dependent manner following chenodeoxycholic acid (CDCA) treatment. **(A)** FGF19 mRNA expression and **(B)** protein levels in cell medium after CDCA treatment. **(C)** Time course of FGF19 mRNA expression (relative to Ctrl) and secreted protein after treatment with a single dose of CDCA. Data in **(A, B)** is presented as box-plot showing interquartile range (IQR) (box) and min-max (bars) with median marked with a line, marked with red dots are the individual values and black dots in **(A)** represents RNA from donors that were also used for RNA sequencing. (24 h, n = 13; 6h, n = 10). The plus sign represents mean value. The time course is presented as median with IQR. Friedman test was used to assess differences between control and treatments and Dunn’s multiple comparison test was used *post-hoc*. *p < 0.05, **p < 0.01, ***p < 0.001.

Given that FGF19 has been described as a main regulator of BA synthesis, the insignificant response of primary hepatocytes to recombinant FGF19 exposure was unexpected. We reasoned that recombinant versus endogenously produced FGF19 can evoke different responses. Previous reports describe low gene expression of FGF19 in untreated primary human hepatocytes, however BAs can induce FGF19 *in vitro* in primary human hepatocytes (9). We first studied CDCA induced FGF19 production by primary human hepatocytes in a dose- and time-dependent manner (**Figure 2**). Our results showed that FGF19 gene expression increased proportionally with increasing concentrations of CDCA (3–20 µM) both 6 or 24 h after treatment when compared to untreated control (**Figure 2A**). In accordance, we detected increasing FGF19 protein secretion with increasing concentrations of CDCA at both time points (**Figure 2B**). These results confirmed that FGF19 gene expression is induced in primary human hepatocytes by CDCA. To resolve temporal dynamics in FGF19 protein secretion, we treated primary human hepatocytes with 10 µM CDCA and quantified FGF19 gene expression and protein levels. We determined that FGF19 gene expression is induced between 1 to 1.5 h after CDCA-treatment and remained constant afterwards. FGF19 protein secretion into the medium commenced with a 2 h delay (between 3 to 3.5 h after CDCA-treatment) (**Figure 2C**).

**Figure 3:**
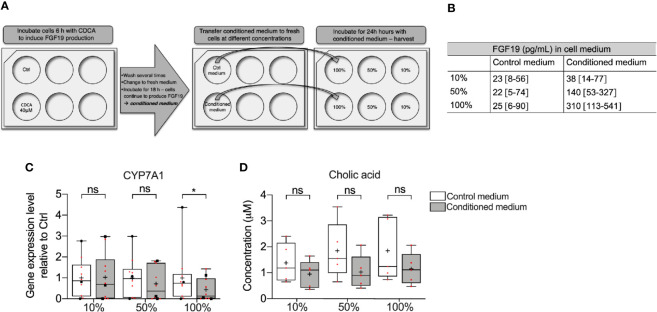
Medium containing FGF19 produced by primary human hepatocytes downregulated CYP7A1 mRNA expression. **(A)** Layout of the experiment showing how chenodeoxycholic acid (CDCA) was used to induce FGF19 synthesis in primary human hepatocytes. Endogenously produced FGF19 was then applied to naïve hepatocytes for 24 h. **(B)** Levels of FGF19 in control and conditioned medium. **(C)** CYP7A1 mRNA expression and **(D)** cholic acid (CA) concentration in control and conditioned medium. Data is presented as box-plot showing interquartile range (IQR) (box) and min-max (bars) with median marked with a line, marked with red dots are the individual values, black dots in **(C)** represents RNA from donors that were also used for RNA sequencing. (n = 10). The plus sign represents mean value. Wilcoxon matched-pairs signed rank test was used to assess differences between control and conditioned medium. *p < 0.05, ns: non significant.

Since CDCA rapidly induced FGF19, we further investigated whether FGF19 endogenously produced by primary human hepatocytes in response to CDCA can downregulate BA synthesis. To assess the effect of endogenously produced FGF19 on CYP7A1 gene expression and CA formation, we treated cells with various concentrations of FGF19 produced by primary human hepatocytes (conditioned medium) for 24 h (**Figure 3A**). We quantified FGF19 protein levels in the conditioned medium to be on average 310 pg/ml, compared to 25 pg/ml in the control medium (**Figure 3B**). We next determined that CYP7A1 gene expression levels remained unchanged when treated with 10% or 50% conditioned medium but was significantly lower in primary human hepatocytes treated with 100% conditioned medium compared to control cultures (**Figure 3C**). Furthermore, we measured CA synthesis and found that the levels of CA between control and conditioned medium did not differ at either concentration (100%; 0.78 µM and 0.70 µM, 50%; 0.97 µM and 0.56 µM, 10%; 1.8 µM and 1.1 µM in control and conditioned medium respectively, **Figure 3D**).

Altogether, CDCA rapidly induced FGF19 in primary human hepatocytes and FGF19 enriched medium downregulated CYP7A1 gene expression. However, as the endogenously produced FGF19 was not purified and the conditioned medium contained traces of CDCA (up to 2 µM, data not shown), further validation of whether it is actually FGF19 that suppresses BA synthesis is needed.

### Treatment of Primary Human Hepatocytes With CDCA Lead to Deregulation of Genes Involved in Metabolic Pathways

**Figure 4:**
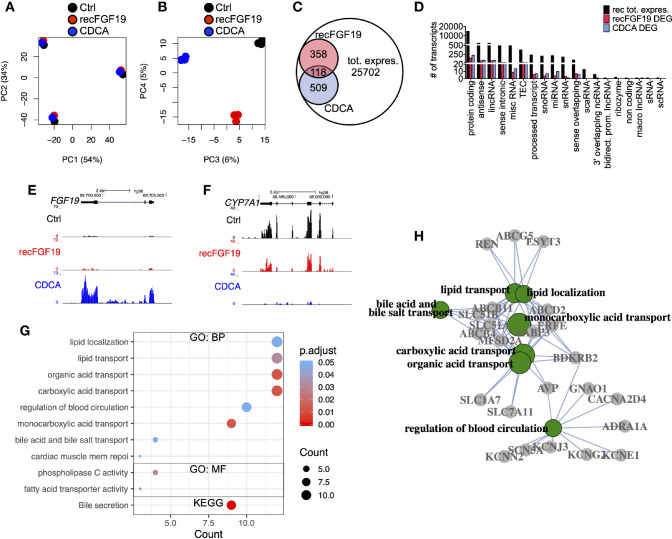
Differentially expressed genes in primary human hepatocytes upon treatment with chenodeoxycholic acid (CDCA) or recombinant FGF19. Total RNA from primary human hepatocytes treated with recombinant FGF19, CDCA, or vehicle control was sequenced (n = 3). **(A, B)** Principal component analysis (PCA) showed that 88% of the variation in the samples could be explained by donor differences (PC1-2), while 11% of the variation could be explained by the treatment (PC3-4). **(C, D)** A limited number of transcripts were differentially expressed upon treatment. Venn diagram **(C)** and bar plot **(D)** displaying all expressed transcripts (black) and differentially expressed ones after recombinant FGF19 treatment (red) or CDCA treatment (blue). **(E, F)** Representative UCSC Genome Browser tracks of normalized FGF19 and CYP7A1 expression after recombinant FGF19 or CDCA treatment. The tracks from top to bottom show the scale in the human genome, the location in the human genome, the gene including exons (black boxes) and introns (arrows), and the RNA-seq signal from each treatment. **(G)** Unique chenodeoxycholic acid (CDCA)-DE transcripts were used for gene ontology (GO) biological processes (BP), molecular function (MF), and KEGG pathway analysis. Displayed are all significant ontologies/pathways in each ontology. The size of the bubble indicates number of genes in each category and the color represents the significance. **(H)** GO-term interaction network of the 7 most significant GO-BP terms in **(G)**. GO terms are in green circles, and gene names are in grey circles. Unique DE transcripts after recombinant FGF19 gave no significant enrichment in GO or KEGG pathway-related terms.

To discern the molecular roles of CDCA and FGF19 on hepatic gene expression, we performed a global analysis of gene expression (**Figure 4**). A principal component analysis (PC) of the experiments performed on primary human hepatocytes treated with 10 µM CDCA or 1,000 pg/ml recombinant FGF19 showed that 88% (PC 1 and 2) of the variation was explained by inter-individual differences given the clustering by donors (**Figure 4A**), while 11% (PCA 3 and 4) of the variation was the consequence of treating primary human hepatocytes with CDCA or recombinant FGF19 (**Figure 4B**). We carried out a differential gene expression analysis and compared each treatment group with untreated controls. Our analysis showed that the vast majority of annotated genes remained unchanged (or highly variable between donors) in primary human hepatocytes but 2.4% and 1.9% of the annotated genes were significantly deregulated (fold-change > ±2, FDR<0.05) upon CDCA and FGF19 treatment, respectively, when compared to untreated controls (**Figure 4C**). We grouped the genes by gene categories, which revealed that both protein coding and noncoding genes were affected upon treatment (**Figure 4D**). CDCA treatment altered the expression of 627 genes (**Figure 4C**, **Table S3**). Among them, we confirmed increased FGF19 (22.7-fold) and decreased CYP7A1 (26.4-fold) gene expression levels (**Figures 4E, F**). In addition, previously described genes involved in BA metabolism (1, 3), such as NR0B2 (encoding the regulatory protein small heterodimer partner [SHP]) was up-regulated (2.7-fold) and the BA transporters SLC51A (OSTα), SLC51B (OSTβ), ABCB11 (BSEP) and ABCB4 (MDR3) were up-regulated (3.1, 17.9, 6.2, and 2.3-fold, respectively) (**Table S3**).

In general, our Gene Ontology (GO) term and KEGG pathway analysis showed that CDCA treatment affected genes involved in lipid and BA transport and secretion (**Figure 4G**, **Table S4**). Categorizing the GO terms showed that many deregulated genes are interconnected (**Figure 4H**). In contrast, a total of 476 transcripts were found differentially expressed upon treatment with recombinant FGF19 when compared to control (**Figures 4C, D**). However, CYP7A1 was not among the differentially expressed genes (**Table S3**). Furthermore, GO term and pathway analyses did not reveal any pathway that was significantly regulated.

**Figure 5:**
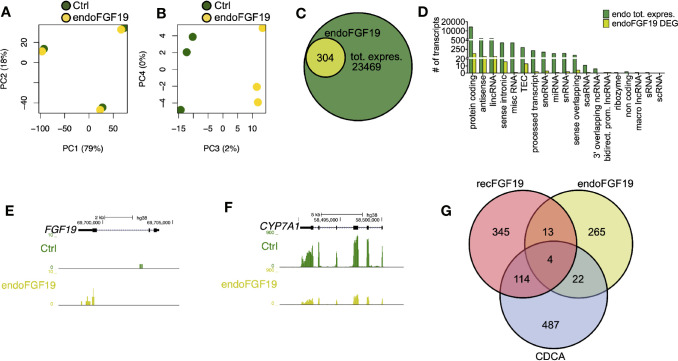
Differentially expressed genes in primary human hepatocytes upon treatment with endogenously produced FGF19. Primary human hepatocytes were treated with endogenously produced FGF19 or vehicle control and total RNA was sequenced (n = 3). **(A, B)** Principal component analysis (PCA) showed that 97% of the variation in the samples could be explained by donor differences (PC1-2), while only 2% of the variation could be explained by the treatment (PC3-4). **(C, D)** A limited number of transcripts were differentially expressed upon treatment. Venn diagram **(C)** and bar plot **(D)** displaying all expressed transcripts (green) and differentially expressed transcripts after endogenously produced FGF19 treatment (yellow). The differentially expressed transcripts were not significantly enriched for any gene ontologies (GOs) or KEGG pathways. **(E, F)** Representative UCSC Genome Browser tracks of normalized FGF19 and CYP7A1 expression after endogenous FGF19 treatment. The tracks from top to bottom show the scale in the human genome, the location in the human genome, the gene including exons (black boxes) and introns (arrows), and the RNA-seq signal from the treatment. **(G)** Venn diagram displaying the overlap between differentially expressed transcripts after chenodeoxycholic acid (CDCA) (blue), recombinant FGF19 (red), or endogenously produced FGF19 (yellow) treatment compared to their respective vehicle controls. All three treatments resulted in mainly unique expressed transcripts, with little overlap among the treatments.

Since recombinant FGF19 had minor effects, we tested the effects of endogenously produced FGF19 on gene expression (**Figure 5**). Similar to the previous treatments, our PCA analysis of cells treated with endogenously produced FGF19 revealed that 97% of the variation in the samples was explained by differences between donors and only 2% by treatment (**Figures 5A, B**). A total of 304 transcripts were differentially expressed when compared to control (**Figures 5C, D**, **Table S3**). As expected, gene expression of CYP7A1 was reduced (6.1-fold) whereas FGF19 and SLC51B – genes normally associated with BA activation of FXR, increased 5.0 and 2.9-fold, respectively (**Figures 5E, F**, **Table S3**). Although these genes were differentially expressed both GO term and KEGG pathway analysis did not give any significant results.

In summary, a number of transcripts were differentially expressed upon the respective treatment and there was little overlap between treatments (**Figure 5G**). Genes involved in BA metabolism were differentially expressed upon treatment with CDCA or conditioned medium containing endogenously produced FGF19 but not with recombinant FGF19.

### CYP7A1 Gene Expression Was Downregulated in Presence of CDCA Irrespective of siRNA-Mediated Knockdown of FGF19

**Figure 6:**
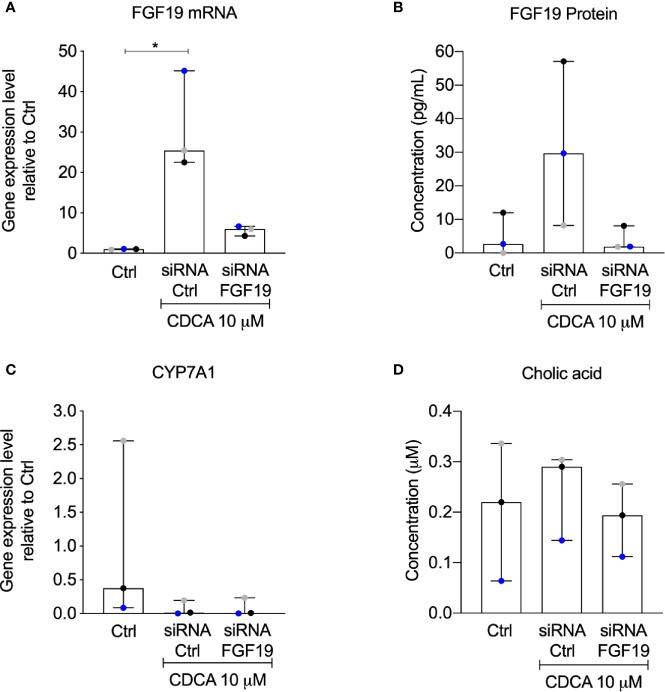
Knockdown of FGF19 did not alter downregulation of CYP7A1 mRNA expression by chenodeoxycholic acid (CDCA). Primary human hepatocytes were treated with siRNA targeting FGF19 or non-targeting control. **(A)** FGF19 mRNA expression, **(B)** protein levels in medium in control cultures, after induction by 10 µM CDCA. **(C)** CYP7A1 mRNA expression, **(D)** cholic acid (CA) concentration in cell medium. Data is presented as median with interquartile range (IQR). Friedman test was used to assess differences between control and treatments and Dunn’s multiple comparison test was used *post-hoc*. Each colored dot represents one individual case (n = 3). *p < 0.05.

The rapid increase of FGF19 by physiological levels of CDCA encouraged us to further validate the contribution of an autocrine pathway for FGF19 regulation of BA synthesis. We therefore performed a knockdown experiment to reduce FGF19 gene expression levels upon CDCA treatment. Following FGF19 siRNA knockdown, we exposed primary human hepatocytes to 10 µM CDCA for 6 h. In accordance to our previous results (**Figure 2**), CDCA induced FGF19 gene expression (**Figure 6A**) and protein secretion into the medium (**Figure 6B**) in primary human hepatocytes transfected with siRNA controls. In contrast, FGF19 gene expression and protein secretion was efficiently reduced upon transfection with siRNA against FGF19 and could not be increased when treated with CDCA (**Figures 6A, B**). In accordance, CYP7A1 gene expression was reduced 43-fold in primary human hepatocytes treated with CDCA when compared to untreated controls irrespective of siRNA-mediated depletion of FGF19 (**Figure 6C**). CA biosynthesis remained unchanged in response to 6 h of CDCA treatment and FGF19 depletion (**Figure 6D**). Our results showed that although CDCA induces FGF19 gene expression and protein secretion in primary human hepatocytes, regulation of BA synthesis in this setting remains intact.

## Discussion

BA homeostasis is regulated by a complex system controlled by BA activated feedback systems. BAs activate nuclear receptors that subsequently induce pathways involving transcription factors and other regulatory proteins to regulate transport, circulation and biosynthesis (1–5). There are different pathways for feedback regulation of BA synthesis in humans, mediated by the BA activated nuclear receptor FXR. The major hepatic pathway involves the orphan nuclear receptor SHP, which function as a transcriptional co-repressor to inhibit CYP7A1 expression (6, 18–21). FGF19 acts as an endocrine molecule, reaching the liver following secretion from intestine, to suppress CYP7A1 by binding and signaling through the fibroblast growth factor receptor 4 (FGFR4)/β-Klotho complex (8, 9, 22). Both of these FXR targets are of importance to maintain BA homeostasis, but as they are both upregulated in response to BAs at the last stages of the enterohepatic circulation, the impact of each of them on CYP7A1 expression and subsequently BA synthesis is difficult to distinguish. This is further complicated by distinct species differences in BA metabolism and conclusions originating from animal models can therefore not be directly translated to humans, which warrants studies on human systems (23–26).

To investigate the hepatic response to FGF19 and CDCA we treated primary human hepatocytes with recombinant FGF19 or CDCA at different concentrations within the physiological range (10, 13, 14). While CDCA downregulated CYP7A1 mRNA expression in a dose-dependent manner, FGF19-treated cells showed no change. RNA sequencing analysis revealed that only a minor subset of transcripts were differentially expressed by recombinant FGF19 treatment. Pathway analyses did not confirm that these were involved in BA metabolism nor was any other pathway significantly affected by recombinant FGF19. Pathway analyses of differentially expressed transcripts from CDCA treated cells, on the other hand, showed genes involved in BA metabolism in agreement with other studies (1, 3). This surprisingly low response by the hepatocytes to recombinant FGF19 has been demonstrated in previous studies where supraphysiologic concentrations of at least 10 times more FGF19 than what is found circulating in humans has been used to see an effect in both animals and cell culture systems (9, 26, 27). The recombinant protein may have properties other than the endogenously produced and/or the biological activity may be different. A study by Kong and Guo (28) suggested that Fgf15, the mouse ortholog of FGF19, is prone to form inclusion bodies when expressed in *Escherichia coli*. They concluded that these aggregates can cause problems with re-folding of the protein *in vitro* subsequent to isolation and purification, which would then render it less biological active. This might be a contributing factor to the lack of effect from recombinant FGF19 and should be important to keep in mind for studies utilizing this synthetic form of FGF19. It should be noted that mRNA expression of the FGF19 receptor, FGFR4, and the co-factor necessary for stabilizing the interaction between FGF19 and its receptor, β-Klotho, were both stably expressed and not affected by treatment (**Supplementary Figure 1**). We designed an experiment to evaluate if the response to endogenous FGF19, produced by the primary human hepatocytes in response to CDCA, differ from recombinant FGF19 and downregulated BA synthesis. Interestingly, we found a dose-dependent decrease of CYP7A1 mRNA expression following treatment with medium enriched with endogenously produced FGF19. However, RNA sequencing analysis of cells treated with conditioned medium also revealed that non-classical FGF19-regulated genes, FGF19 and SLC51B that are both direct targets of CDCA-activated FXR (1, 27), were also among the differentially expressed genes. Upregulation of these genes could thus be explained by trace amounts of CDCA remaining in the conditioned cell medium. Indeed, BA analysis revealed CDCA levels of up to 2 µM in the conditioned medium (data not shown). Further validation of purified endogenously produced FGF19 and how it affects bile acid synthesis is thus needed.

FGF19 mRNA and protein expression in primary human hepatocytes was upregulated by CDCA in a dose-dependent manner. Interestingly, this occurred rapidly after treatment and with a dose of CDCA within the physiological range (14). FGF19 mRNA expression started increasing after 1 h followed by protein secretion after about 2 h. The rapid induction of FGF19 and the release into the cell medium is in agreement with the time frame of the postprandial peak of FGF19 observed in plasma that follows the peak of BAs. This suggests that FGF19 is potentially upregulated in liver also under physiological conditions, although it is unclear if FGF19 produced *in vivo* under normal circumstances would be enough to affect BAs synthesis. Therefore, to further evaluate the impact of hepatocyte produced FGF19 on BA synthesis, we conducted a knockdown experiment of FGF19. Following knockdown of FGF19 and after CDCA induction we found that FGF19 levels did not differ from the control cultures. Knockdown of FGF19 gene expression in primary human hepatocytes had no significant effect on CYP7A1 expression, which was still successfully downregulated 43-fold by CDCA, presumably *via* the previous described FXR-SHP-pathway (6). Song et al. (9) showed that when using an antibody against FGF19 or silencing FGFR4 in primary cell cultures in combination with the synthetic FXR agonist GW4064 the expression of CYP7A1 is increased, compared to cultures that were only treated with GW4064. However, from the data presented in Song et al. CYP7A1 is still downregulated in cultures treated with GW4064 regardless of antibody/siFGFR4 treatment when compared to cultures treated with DMSO vehicle control, which would be in support of the data in our study (9). We speculate that the upregulation of hepatic FGF19 is not crucial for maintaining BA homeostasis under normal conditions but that this may become of importance under conditions (*e.g.* cholestasis) when the liver experience excess levels of BAs and when the liver is not receiving FGF19 from the intestine (11, 12, 29). A possible explanation for the rapid response of FGF19 to CDCA has been suggested to be in the stabilization of SHP (30). SHP is rapidly degraded *via* the ubiquitin-proteasomal pathway. Studies have shown that FGF19 increase phosphorylation of extracellular signal-regulated kinase (ERK), which in turn dramatically decrease ubiquitination and proteasomal degradation of SHP. Inhibition of ERK on the other hand result in a dramatic reduction of SHP, suggesting that FGF19-mediated activation of ERK could be of importance for SHP stability and a possible autocrine or paracrine function of FGF19 in regulation of BA homeostasis (9, 30).

In summary, we have shown that levels of CDCA compatible with postprandial levels in portal blood downregulated bile acid synthesis and rapidly upregulated FGF19 expression in primary human hepatocytes. Downregulation of BA synthesis by CDCA is independent of endogenously produced FGF19.

## Data Availability Statement

The RNA-seq dataset generated during the current study is available in the ArrayExpress repository, under accession number: E-MTAB-8627 (https://www.ebi.ac.uk/arrayexpress/experiments/E-MTAB-8627).

## Ethics Statement

The studies involving human participants were reviewed and approved by Swedish ethics review authority in Stockholm (Dnr:2017/269-31). The patients/participants provided their written informed consent to participate in this study.

## Author Contributions

Study design by EE, HJ, CK, and JNS. Sample and data collection by HJ, JNS, and CJ. Writing manuscript by EE, HJ, CJ, CK, JNS. Data analysis by EE, HJ, CJ, CK, JNS. All authors contributed to the article and approved the submitted version.

## Funding

Knut & Alice Wallenberg foundation (KAW 2016.0174, CK), Ruth & Richard Julin foundation (2017–00358, 2018–00328, 2020-00294 CK); Swedish Research Council (2019–05165, CK), SNIC projects (2017/7-154 and sllstore2017022, JNS, CK), Nilsson-Ehle Endowments (JNS).

## Conflict of Interest

The authors declare that the research was conducted in the absence of any commercial or financial relationships that could be construed as a potential conflict of interest.

## Supplementary Material

Supplementary Figure 1 FGFR4 and βKlotho was not affected by FGF19 or CDCA treatment. **(A, B)** FGFR4 mRNA expression and **(C, D)** βKlotho mRNA expression following treatment with FGF19 or CDCA at various concentrations 6 h (n = 10) or 24 h (n = 13). Data is presented as box-plot showing IQR (box) and min-max (bars) with median marked with a line, red dots are the individual values. The plus sign represents mean value. Friedman test was used to assess differences between control and treatments (all non significant).

Supplementary Table 1 htseq counts Chr1-22.

Supplementary Table 2 TMM values.

Supplementary Table 3 DEG analysis results.

Supplementary Table 4 GO pathway analysis.

## Abbreviations

CA, cholic acid; CDCA, chenodeoxycholic acid; CYP7A1, cholesterol 7-alpha hydroxylase; ERK, extracellular signal-regulated kinase; FGF19, fibroblast growth factor 19; FGFR4, fibroblast growth factor receptor 4; FXR, Farnesoid X receptor; SHP, small heterodimer partner.
