# Supplementary figures and images for: Chenodeoxycholic Acid Modulates Bile Acid Synthesis Independent of Fibroblast Growth Factor 19 in Primary Human Hepatocytes
Source: Front Endocrinol (Lausanne). 2021 Feb 22;11:554922. doi: 10.3389/fendo.2020.554922 (PMC7937932; doi:10.3389/fendo.2020.554922)

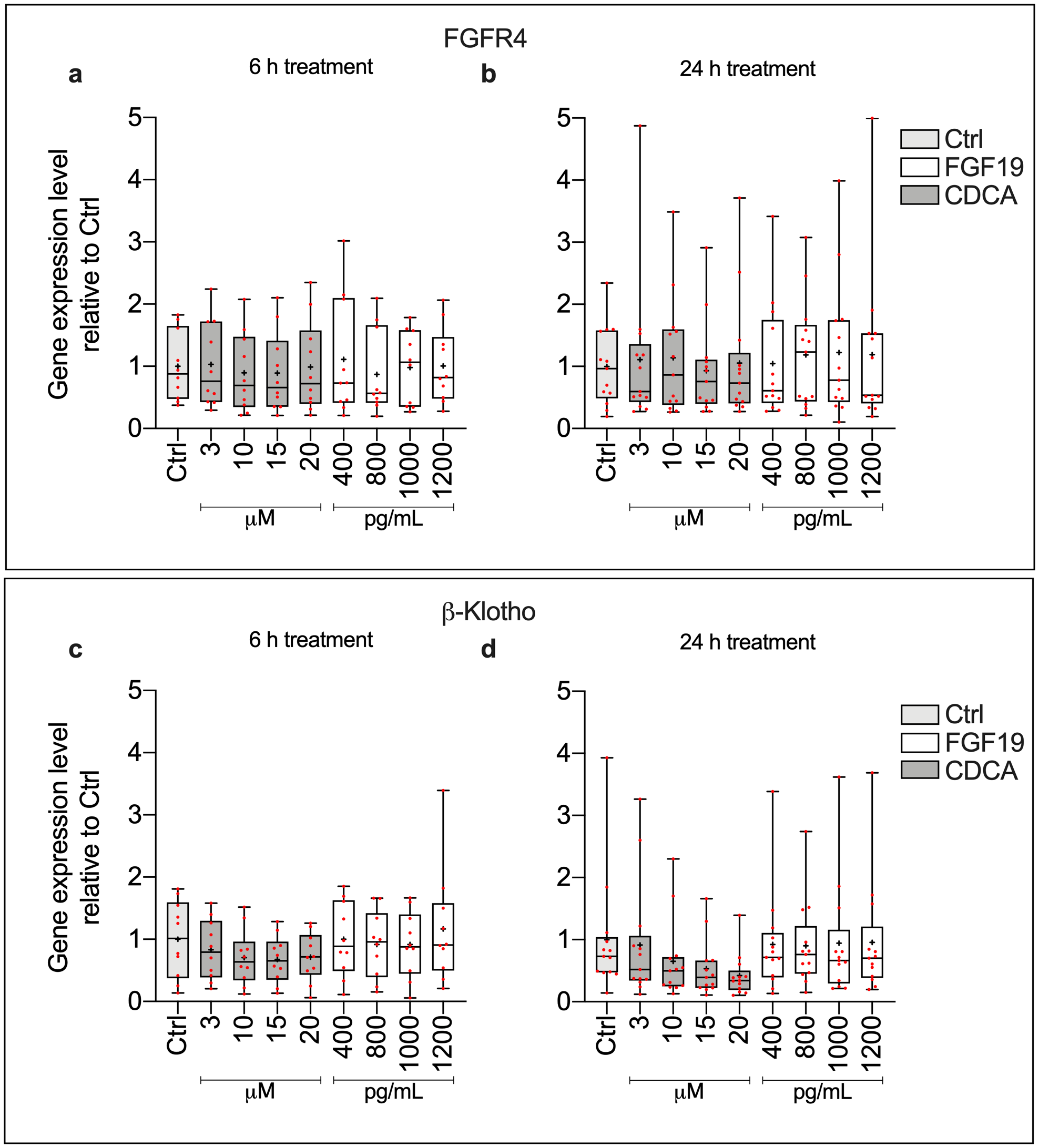

Supplement: Supplementary Figure 1 — FGFR4 and βKlotho was not affected by FGF19 or CDCA treatment. (A, B) FGFR4 mRNA expression and (C, D) βKlotho mRNA expression following treatment with FGF19 or CDCA at various concentrations 6 h (n = 10) or 24 h (n = 13). Data is presented as box-plot showing IQR (box) and min-max (bars) with median marked with a line, red dots are the individual values. The plus sign represents mean value. Friedman test was used to assess differences between control and treatments (all non significant). [file Image_1.tiff]
